# Going beyond the means: Exploring the role of bias from digital determinants of health in technologies

**DOI:** 10.1371/journal.pdig.0000244

**Published:** 2023-10-12

**Authors:** Marie-Laure Charpignon, Adrien Carrel, Yihang Jiang, Teddy Kwaga, Beatriz Cantada, Terry Hyslop, Christopher E. Cox, Krista Haines, Valencia Koomson, Guillaume Dumas, Michael Morley, Jessilyn Dunn, An-Kwok Ian Wong

**Affiliations:** 1 Massachusetts Institute of Technology; Institute for Data, Systems, and Society; Laboratory for Information and Decision Systems, Boston, Massachusetts, United States of America; 2 CentraleSupélec, Université Paris-Saclay, Gif-sur-Yvette, France; 3 Imperial College London, London, United Kingdom; 4 Duke University, Pratt School of Engineering, Department of Biomedical Engineering, Durham, North Carolina, United States of America; 5 Mbarara University of Science and Technology, Department of Ophthalmology, Mbarara, Uganda; 6 Massachusetts Institute of Technology; Institute Community and Equity Office, Boston, Massachusetts, United States of America; 7 Duke University, Department of Biostatistics and Bioinformatics, Durham, North Carolina, United States of America; 8 Duke University, Department of Medicine, Division of Pulmonary, Allergy, and Critical Care Medicine, Durham, North Carolina, United States of America; 9 Duke University, Department of Surgery, Durham, North Carolina, United States of America; 10 Tufts University, Department of Electrical and Computer Engineering, Boston, Massachusetts, United States of America; 11 CHU Sainte-Justine Research Center, Department of Psychiatry, Université de Montréal, Montréal, Quebec, Canada; 12 Mila–Quebec AI Institute, University of Montreal, Montréal, Quebec, Canada; 13 Ophthalmic Consultants of Boston, Boston, Massachusetts, United States of America; 14 Assistant Clinical Professor of Ophthalmology, Harvard Medical School, Boston, Massachusetts, United States of America; St Luke’s Medical Center, PHILIPPINES

## Abstract

**Background:**

In light of recent retrospective studies revealing evidence of disparities in access to medical technology and of bias in measurements, this narrative review assesses digital determinants of health (DDoH) in both technologies and medical formulae that demonstrate either evidence of bias or suboptimal performance, identifies potential mechanisms behind such bias, and proposes potential methods or avenues that can guide future efforts to address these disparities.

**Approach:**

Mechanisms are broadly grouped into *physical and biological biases* (e.g., pulse oximetry, non-contact infrared thermometry [NCIT]), *interaction of human factors and cultural practices* (e.g., electroencephalography [EEG]), and *interpretation bias* (e.g, pulmonary function tests [PFT], optical coherence tomography [OCT], and Humphrey visual field [HVF] testing). This review scope specifically excludes technologies incorporating artificial intelligence and machine learning. For each technology, we identify both clinical and research recommendations.

**Conclusions:**

Many of the DDoH mechanisms encountered in medical technologies and formulae result in lower accuracy or lower validity when applied to patients outside the initial scope of development or validation. Our clinical recommendations caution clinical users in completely trusting result validity and suggest correlating with other measurement modalities robust to the DDoH mechanism (e.g., arterial blood gas for pulse oximetry, core temperatures for NCIT). Our research recommendations suggest not only increasing diversity in development and validation, but also awareness in the modalities of diversity required (e.g., skin pigmentation for pulse oximetry but skin pigmentation and sex/hormonal variation for NCIT). By increasing diversity that better reflects patients in all scenarios of use, we can mitigate DDoH mechanisms and increase trust and validity in clinical practice and research.

## Introduction

Novel medical technologies have arisen to assist clinical teams and facilitate diagnosis by physicians, especially under budget constraints: Since 2010, 523 new medical devices have been approved for commercialization by the Food and Drug Administration (FDA). In parallel with this development, retrospective studies have revealed evidence of disparities in access to medical technology and of bias in the measurements resulting from such devices.

While previous articles have focused on *social and economic* determinants of health, this narrative review investigates *digital* determinants of health, as defined earlier in this collection [1]. Specifically, it identifies digital technologies and medical formulae that demonstrate evidence of bias or suboptimal performance. Such pitfalls generally arise from insufficient consideration of patient diversity. Herein, we describe some known physical or biological mechanisms underpinning differences among patients—including those based on sex (either current or at birth), race, and ethnicity—and identify ways in which these characteristics affect the accuracy of digital medical technology for some populations. One such example is pulse oximetry: disparities in its performance among racial groups are thought to result from a lack of patient diversity in clinical trials [**2**]. Another example is body temperature measurement: Differential thermoregulation among females affects the estimates provided by some thermometers. Further, we explain possible repercussions of these biases on digital determinants of health, formulate potential reasons why inadequate patient sampling has resulted in such impacts, and derive implications for clinical care. Finally, when applicable, we present existing solutions to mitigate these biases or suggest ways that corrections may be developed.

This review does not cover the impact of medical technologies relying on artificial intelligence and machine learning, such as algorithms for clinical decision-making. Indeed, insufficient diversity in patient sampling—commonly due to selection bias, inequitable decision-making, or systemic racism—has already been well documented as influencing the performance of these models [3]. This review also excludes the direct impacts of *social* determinants of health, such as the underdetection of diabetes among patients of color due to factors affecting their access to medical care, which is also a topic well documented in the literature.

Based upon the framework by Kadambi and colleagues [4], we organize this review as follows. First, we describe biases based on characteristics that patients were born with and that are immutable without active intervention, e.g., biological sex at birth or skin tone (*Physical and biological bias* section). Then, we transition to discuss biases that can result from the confluence of medical technology and community-dependent cultural or social norms, e.g., the quality of electroencephalography (EEG) signals may vary with patient hairstyles (*Interaction of human factors and cultural practices* section). Finally, we consider biases resulting from design choice or interpretation (e.g., formulae for pulmonary function tests (PFTs) including race as a factor, although valid alternatives excluding it have been proposed) (*Interpretation bias* section).

### Definitions

Some terms used to characterize the 3 types of bias listed above have inconsistent definitions. As a preamble, we define the terms *skin tone*, *calibration*, and *discrimination*.

This paper uses the terminology *skin tone* instead of *skin color* to describe the color of the skin, including concepts of melanin concentration along with jaundice and other skin pigments. We believe this framing choice is essential as the term is more nuanced and inclusive, including all gradients of skin pigmentation.

Moreover, we reference Alba and colleagues in definitions of calibration and discrimination [5]. Calibration refers to the accuracy of absolute estimates, effectively comparing empirical observations with predicted or estimated measurements (e.g., arterial blood gas oxygen saturation versus pulse oximetry) and improving their correlation by adjusting device settings. Discrimination refers to how well a model can differentiate between groups.

We also use the standard terminology of verification, analytic validation, and clinical validation following the V3 Framework [6].

## Physical and biological bias

The characteristics of a patient’s skin can influence the performance of medical devices, as illustrated by BioMetric Monitoring Technologies (BioMeTs). Pulse oximetry and non-contact infrared thermometry (NCIT) provide 2 such examples.

### Pulse oximetry

Pulse oximetry is a common device that measures oxygen saturation or SpO_2_. Physiologically, pulse oximeters compare absorption at 2 wavelengths of light to estimate the ratio between deoxyhemoglobin and oxyhemoglobin in arterial blood, thereby performing a simplified version of spectrometry [7].

**Fig 1:**
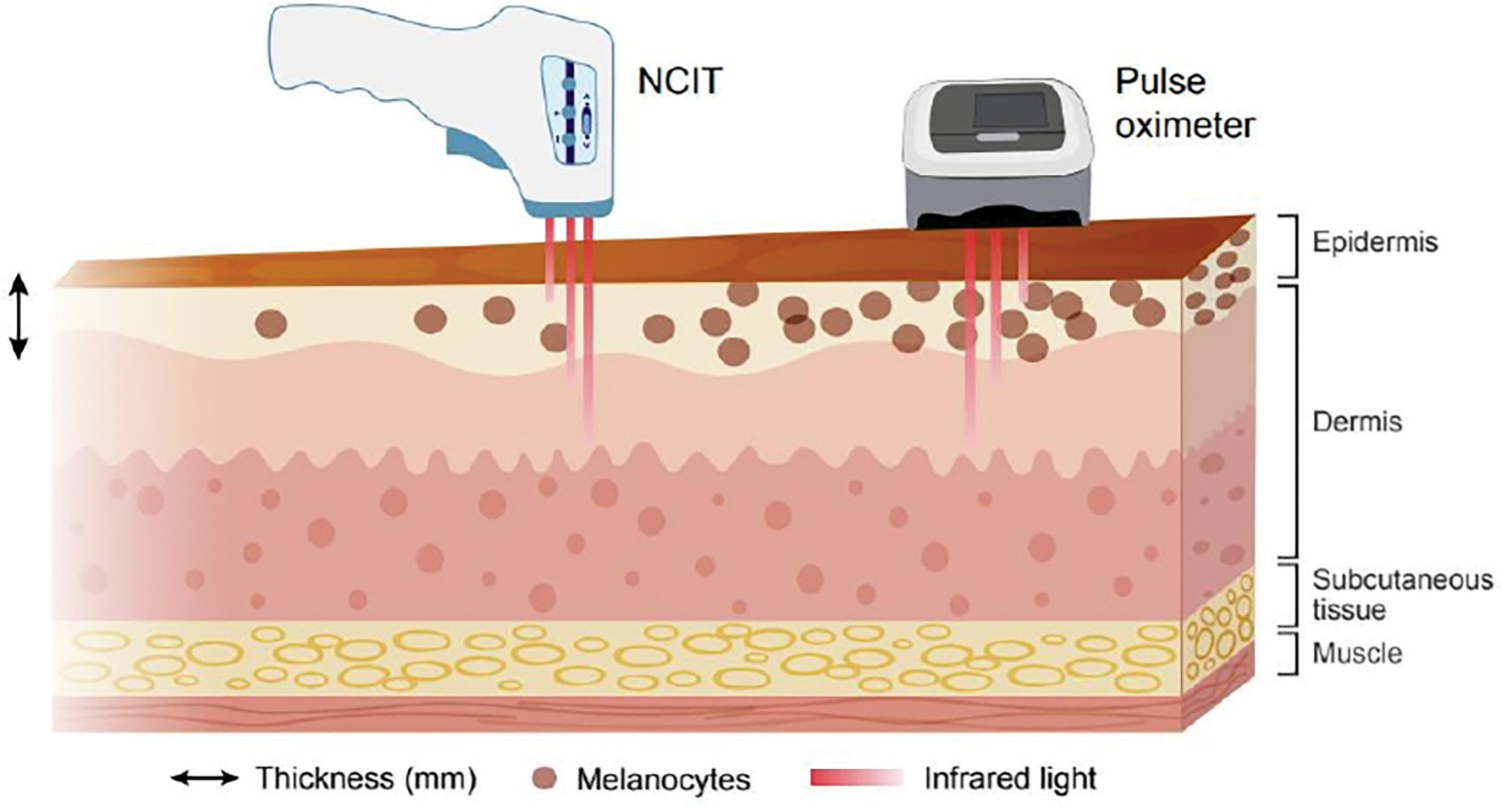
Differential light absorption as a function of melanin levels and skin thickness. Alone or in combination, these factors can alter the performance of medical devices relying on red and/or infrared light. NCIT, non-contact infrared thermometry.

Differential performance of pulse oximetry across patient subpopulations has been known for over 4 decades [8–12] but has recently been brought back to the forefront due to large-scale data analyses by Sjoding and colleagues [13], Wong and colleagues, and Henry and colleagues [13–15], suggesting persisting racial-ethnic disparities in oxygen readings. Despite being debated, evidence suggests that pulse oximeters overestimate actual oxygen levels in hospitalized and intensive care unit (ICU) patients, especially at lower oxygen saturations [16]. Oxygen saturation measurements may be influenced by melanin (Fig 1), a chromophore of the skin present in higher concentrations in patients of darker skin tone that affects light absorption—a key element underlying this technology [7]. This artifact appears to be the likely mechanism underpinning disparities in the performance of pulse oximeters among racial-ethnic subgroups [8,9].

In the United States, the FDA, which regulates medical devices and ensures their safety and effectiveness, requires at least 2 individuals or >15% of patients participating in trials evaluating new pulse oximeters to have “darkly pigmented skin.” Yet, clear guidance on how skin pigmentation should be quantified or measured is still lacking [17]. Results from the large studies by Sjoding, Wong, and Henry [13–15] demonstrate persisting limitations, with heterogeneous mean absolute percentage errors in the estimation of blood oxygen saturation across racial-ethnic subgroups, reemphasizing that FDA requirements were insufficient to ensure appropriate calibration of pulse oximeters. In response to the publication by Sjoding and colleagues, the FDA released a warning to raise awareness among clinicians about the potential lower accuracy of pulse oximeters for patients with darker skin. However, the agency currently provides no specific recommendations to counteract such measurement biases in device evaluation or in clinical practice [18].

Several approaches might address the lack of systematic device evaluation. First, similar to the performance of other medical devices, the evaluation of pulse oximeters can be improved by enrolling more patients in clinical trials and by ensuring a more diverse set of patients, i.e., with a variety of skin tones. Determining the optimal overall sample size and sociodemographic composition of a trial can be challenging. This reality warrants more methodological work to guide power analysis by anticipating effect sizes and calculating adequate population size and distribution across patient strata. Meanwhile, case studies of a few thousand patients would be an acceptable option. Beyond enhancements in study design and data collection, better representation of patients from different racial and ethnic origins should be sought. Despite guidelines from the National Institutes of Health (NIH) advocating for better representation of communities of color in clinical research [19,20] and similar commitments by the FDA Office of Minority Health and Health Equity [21], barriers to their enrollment remain at multiple levels: (a) systemic (e.g., community hospitals lacking the infrastructure to support clinical trials, despite capturing a more diverse population); (b) individual (e.g., the reluctance of healthcare professionals to register patients from underrepresented racial-ethnic communities due to implicit bias of oft-speculated lower adherence to assigned treatment); and (c) interpersonal (e.g., doctor–patient relationship and building of trust required for a patient to accept to join a trial). Moreover, some patients may have a historically motivated mistrust of the research enterprise associated with violating their human rights in the past [22,23]. In America, the *All of Us* program launched by the NIH in 2018 was the first step toward improved patient representation. Expected to continue for at least a decade, this study aims to collect data from over 1 million people of different racial-ethnic origins, ages, and backgrounds who live in all parts of the country [24]. Since measurement inaccuracies are thought to be more prevalent among individuals with darker skin [8,9,13–15], it may be relevant to oversample patients across a gradient of darker skin tones when recruiting for studies. Going forward, computational modeling will be key to enhancing study population design by predicting likely effect size ranges via simulations. For example, tissue-mimicking phantoms that closely reproduce the properties of human tissue can be leveraged to elaborate on existing medical devices or propose new treatment options [25]. These bench-top methods are already used in optics to understand the optical characteristics of biological tissues, standardize bio-optical techniques, and calibrate metrics on human-like tissues before issuing a clinical trial [26]. Similarly, quantitative biology and pharmacology studies increasingly rely on interconnected microphysiological systems or organs-on-chips [27]. Using a feedback loop process, these in vitro studies can be tested experimentally and results of in vivo tests integrated in the simulation pipeline through iterative updates.

Second, by learning from the discrepancies observed among patients of a given skin tone using paired arterial blood oxygen saturation (SaO_2_) and pulse oximetry (SpO_2_) measurements, statistical solutions could potentially be developed to de-bias raw measurements from the pulse oximeter. One way to address these first 2 approaches could include estimating weightings for measurement value adjustment as a function of skin tone, age, and other patient characteristics, and then applying them as part of a correction formula. To our knowledge, existing devices do not currently implement such a strategy.

Third, new pulse oximeter architectures are being developed to address the numerous noise sources in the photoplethysmography (PPG) waveform, forming the basis for SpO_2_ measurement. The PPG signal is affected by individual user variations (e.g., skin tone, skin thickness, body mass index (BMI), age, temperature, perfusion index, and sex) and environmental perturbations (e.g., motion artifact, sensor placement). Several studies have shown the use of polarized imaging-based techniques to discriminate between light components reflected from various penetration depths to suppress skin effects and improve SpO_2_ accuracy [28,29]. Such architectures may reduce inaccuracies in oxygen saturation measurements and ensure similar calibration across subpopulations. Further, these 3 solutions—from revised clinical trial cohort composition principles to the estimation of statistical learning-based correction terms and improved device design—could be combined to allow their respective effects to compound. All recommendations for pulse oximetry have been summarized in Table 1.

**Table 1:** Summary for pulse oximetry recommendations.

Technology	Recommendation	Strength	Level of evidence
Pulse oximetry	Clinical: Pulse oximetry may be less accurate, especially as oxygen saturations decrease and in patients of color. Consider confirmatory testing with arterial blood gas and potentially modifying oxygen therapy.	B	III
	Clinical: Interventions requiring specific oxygen thresholds may need to be reevaluated, potentially across skin tones.	E	V
	Research: Prospective trials should incorporate sufficient diversity among participants to identify differential rates of deleterious outcomes, such as patient status deterioration, increased length of stay, or mortality.	E	V
	Research: Outcomes defined using pulse oximetry in research trials may need another method to measure oxygenation.	E	V

### Non-contact infrared thermometers (NCITs) and temporal artery thermometers (TATs)

As a vital sign, body temperature is routinely monitored in hospital settings. It is generally used to assess health status, facilitate diagnosis, and target treatments [30,31]. Given the widespread use of non-contact infrared thermometers (NCITs) during the Coronavirus Disease 2019 (COVID-19) pandemic to detect fever associated with Severe Acute Respiratory Syndrome Coronavirus 2 (SARS-CoV-2) infection [32], the evaluation of potential racial and ethnic biases in the performance of such devices has reemerged. There is a precedent for using NCITs in emergency settings. For example, NCITs have already been used to screen for fever during past epidemics, including SARS in 2003 and H1N1 in 2009 [33–35]; they are also currently recommended as a useful screening device for prevention in the FDA’s COVID-19 pandemic guidelines [36].

Despite widespread adoption of NCITs in response to the COVID-19 pandemic, evidence comparing the performance of NCITs with that of devices commonly used for temperature measurement in adults is lacking. This prompted Australian researchers in May 2021 to study the difference between temperature measurements taken by NCITs and temporal artery thermometers (TATs)—considered as a gold standard device for inpatient care in Australian hospitals [31]. Both devices use infrared sensors and estimate body temperature from skin temperature measurements. However, patient characteristics such as skin tone and biological sex can affect the accuracy of temperature measurements [31].

**Fig 2:**
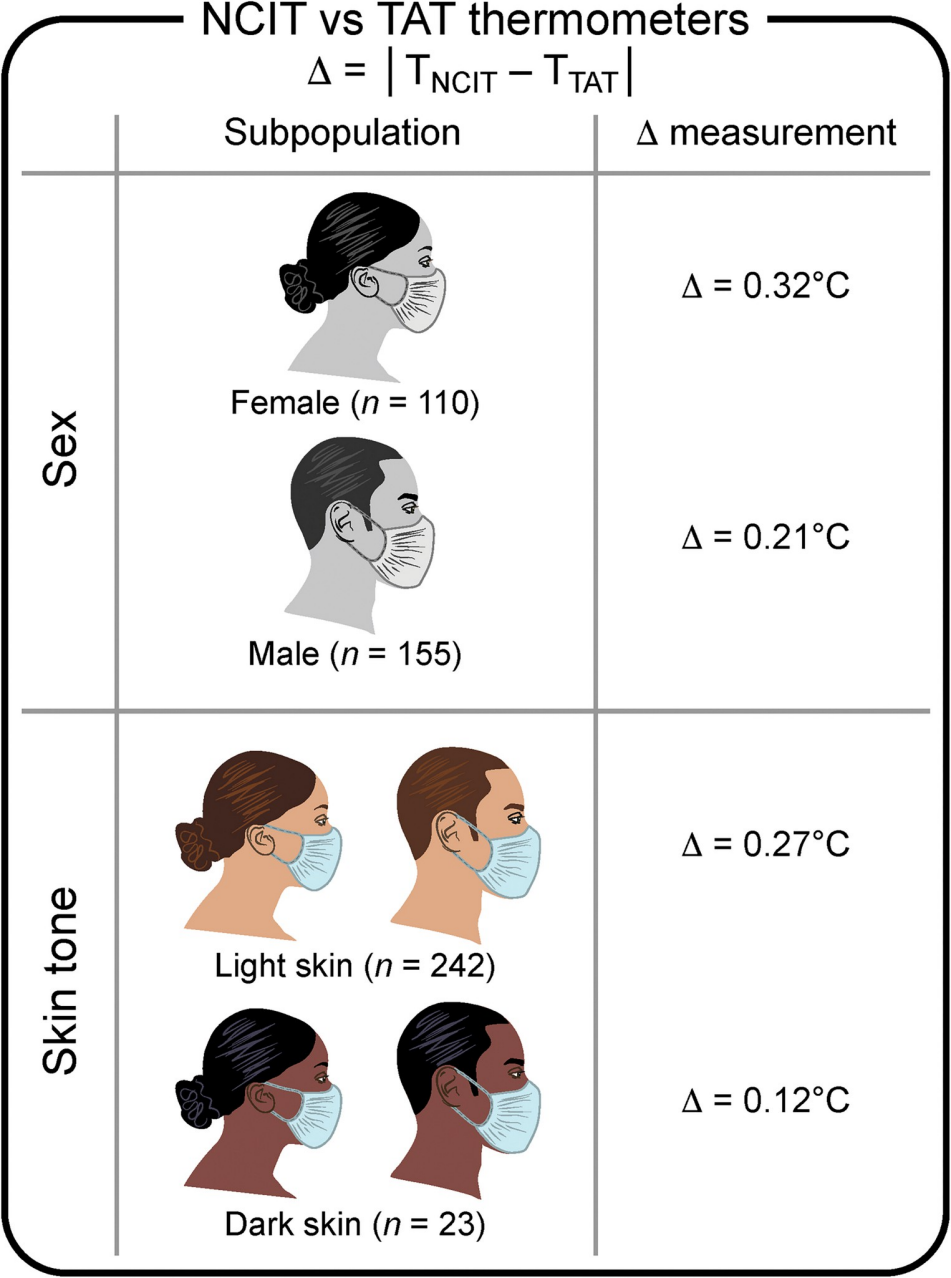
This figure describes the discrepancy between NCIT and reference TAT measurements. **Source:** Data from [31] Khan and colleagues, Comparative accuracy testing of non-contact infrared thermometers and temporal artery thermometers in an adult hospital setting. Am J Infect Control. 2021. NCIT, non-contact infrared thermometry; TAT, temporal artery thermometer.

Overall, NCITs were less precise than reference TATs, as measured by the absolute mean difference between measurement types [31] (Fig 2). Specifically, according to the Australian study, patients with light skin tone had a larger difference between body temperature estimates resulting from the 2 devices (0.27°C) than those with medium dark skin tone (0.12°C). Additionally, NCIT demonstrated a larger difference in females (0.32°C) than in males (0.21°C). In contrast with other medical devices, where inaccuracies mostly arise in darker-skinned individuals, the lack of instrument precision affecting NCITs—as estimated by the absolute mean difference with the reference measurement—was higher for light-skinned individuals.

Of clinical importance, the difference in body temperature estimates derived from the 2 thermometer types was larger when the actual body temperature was higher than 37.5°C (99.5°F). In these circumstances, using an NCIT rather than a TAT could lead to an incorrect diagnosis since most healthcare providers consider a patient to have a fever when their body temperature exceeds 38°C (100.4°F) [37]. Although these deviations may seem small, the normal body temperature only ranges from 36.16°C to 37.02°C (97.1 to 98.6°F); therefore, the observed differences based on skin tone and sex represent up to 37% of the overall healthy range of body temperatures [38]. Given this tight interval of temperature values, a deviation of up to 0.5°C can span up to half of this range. Similarly, in another recent retrospective study [39], the use of temporal rather than oral temperature measurements consistently yielded a lower likelihood of identifying fever in Black patients, irrespective of the considered temperature cutoff, while no such difference was found in White patients.

In women, temperature fluctuations due to hormone cycles can prevent reliable comparison of temperature measurements over time and thus further complicate patient evaluation and subsequent treatment decision-making. Indeed, the luteal phase of the menstrual cycle (and high-hormone phases in women using oral contraceptives) is associated with an increase in body temperature by 0.5°C [40,41]. In parallel, prior research has shown that females show greater thermal responses to exogenous and endogenous heat loss than males—a likely cause of mismeasurements in body temperature [42]. This influence could affect the infrared energy measured by these devices. Therefore, the inaccuracy of temperature measurements for a given individual may be subject to time-varying perturbations, e.g., during different phases of the hormonal cycle. This reality could have important implications in medical practice. For example, a clinician monitoring a female patient with COVID-19 may not reliably track the status of their patient as daily changes in estimated body temperature [43]. This difficulty can result from either underlying device inaccuracy, hormone-induced fluctuations, or the combination of both factors—making causal interpretation challenging. In summary, a negative difference in estimated basal body temperature measurements could lead to underdiagnosis (false negative). Conversely, a positive difference could potentially lead to overdiagnosis (false positive).

Existing research suggests that the design of thermometers may have contributed to such discrepancies: the intrinsic properties of a patient’s skin (e.g., tone, thickness, perspiration) [31], likely to affect estimated body temperature measurements, may not have been considered by manufacturers while calibrating the device.

Going forward, improving the sensitivity-specificity trade-off of NCITs will thus require elaborating patient-specific adjustment factors for temperature. In particular, further studies are needed to determine the influence of other patient-related factors—such as age, blood flow under the skin, metabolic rate, cardiac output, and hormonal levels—on the accuracy and reliability of temperature measurements. This step will be crucial to mitigating the thermometer’s inaccuracy in women and individuals with varying skin tones. Moreover, more research should be dedicated to characterizing intersectional sources of bias, e.g., biological sex and skin tone, associated with this medical technology to remediate improper device evaluation. To date, no dataset has a sufficiently large sample size to quantify differences between temperature measurements emanating from NCITs and TATs, stratified by biological sex and skin tone. However, multiple sources of bias could compound in practice: Although there currently is no evidence for this yet, device discrepancies associated with more pronounced thermal responses could add to those related to skin tone, yielding higher rates of inaccurate body temperature estimates, for example, among females with fever and with a lighter skin tone. Adequate documentation and reporting of the characteristics of patients enrolled in prospective studies and trials evaluating thermometers should be emphasized. Without such documentation, quantifying digital determinants of health, monitoring their temporal evolution, and correcting for addressable biases in measurements of both body temperature and other vital signs will be infeasible. All recommendations for NCIT have been summarized in Table 2.

**Table 2:** Summary for NCIT recommendations.

Technology	Recommendation	Strength	Level of evidence
NCIT	Clinical: Use of non-contact infrared thermometers may demonstrate wider variability in temperatures above 37.5°C, among patients with lighter skin pigmentation, and among females. Correlate clinically or with another modality.	B	III
	Research: Menstrual cycles are associated with substantial temperature variations in females. Further research in females should consider the effect of these temperature variations on thresholds.	E	V

NCIT, non-contact infrared thermometry.

### Understanding more about the relationship between skin tone and DDoH

More generally, the issues seen in the case of pulse oximeters and NCITs may be encountered in other non-invasive medical technologies that involve light and in particular infrared light. Optical techniques routinely used in healthcare rely heavily on absorption and scattering properties of light in human tissue, which depend on the skin’s thickness and the density of chromophores such as melanin, among other factors [44–46] (Fig 1). Because melanin levels determine skin tone, incorporating the latter as an adjustment factor is critical when developing medical devices. Given the complexity of skin tone gradients among patients—within and across races, ethnicities, anatomical sites [47], geographies, and cultures, using a continuous variable for skin tone seems ideal. Yet, performing this kind of measurement may be practically inconvenient and more time-consuming than self-reporting or categorical classification based on human perception, especially when the trial is sizable. Furthermore, in trial settings, transparent reporting of the composition of patient cohorts and the performance of medical devices may be more challenging if adopting a continuous scale for skin tone.

While most past trials only stratified patients into 2 groups (namely, light versus dark skin tone) due to practical or sample size considerations, more recent studies use the Fitzpatrick scale to categorize patients into 6 different skin tone categories. However, the Fitzpatrick scale was created to estimate the response to ultraviolet exposure across skin types in dermatological research and was not intended for medical device testing. Additionally, it was developed based on a patient cohort that only included Caucasians [48]. With only 2 categories capturing darker skin tones, the Fitzpatrick scale does not equally represent all racial subgroups and may thus lead to biases when validating medical devices. To address the issue of the limited number of skin tone categories, other scales have been developed, including the CIELAB system (1976), CIECAM02 (2002), and, more recently, the Monk skin tone scale [49]. Interestingly, although the 3D CIELAB framework was not specifically designed for dermatology but instead part of a larger initiative to standardize color ordering systems, it was later found to correlate skin tone well, with 1 parameter capturing pigmentation level and the 2 others defining chroma and hue. In contrast, the Monk scale was intentionally created by sociologist Dr. Ellis Monk in partnership with Google Research to provide a more inclusive spectrum of skin tones. Now incorporated into Google’s products, it can be leveraged to enhance representation and labeling in computer vision datasets and improve the evaluation of machine learning models concerning fairness metrics. Historically, attempts to define richer skin tone scales and colorimetric color spaces beyond dermatology had been made prior to Fitzpatrick’s research. In the early 1900s, von Luschan had proposed the 36-category scale subsequently used in race studies and anthropometry, while Munsell created the general-purpose 3-category colorimetric color space later selected by the US government for soil and geological research. Yet, they presented some pitfalls, including the inconsistency of their measurements at the time based on human perception. In the continuity of these pioneering inventions, advancements in computer vision and recognition systems made over the past decade have alleviated the limitations of human perception and unlocked the use of broader scales. However, using richer skin tone scales and reporting medical device performance based on such a spectrum rather than on discrete categories may require larger patient study cohorts. Furthermore, standardized skin tone scales alone are insufficient. While technologies are now available to more objectively assign a continuous numerical value corresponding to the skin tone of a given individual [50], their adoption in research and clinical practice would benefit from policies imposing the inclusion of a more representative set of skin tones in prospective studies. For example, objective skin tone measurements can be obtained using reflectance spectrometry with multiple wavelengths or cutaneous colorimeters based on general-purpose color systems such as the CIE color space. Spectrophotometers measure the transmittance and reflectance of light through a medium as a function of wavelength in all regions of the electromagnetic spectrum. In contrast, colorimeters measure absorbance, i.e., the extent to which a medium absorbs a specific color of visible light. Since cutaneous colorimeters measure the skin tone by reflecting the absorbed wavelength into trichromatic filters of red, green, and blue, subjective biases owing to arbitrary boundaries of skin tone categories set by humans can be avoided. Most importantly, the self-reporting of skin tone should be strictly limited. Instead, NIH and FDA policymakers should establish and enforce a standardized method to measure skin tones. In addition to promoting the harmonized measurement of skin tone, regulations could more closely monitor the release of medical technologies relying on light to ensure that they are not biased towards patients with specific skin tones. On the manufacturing side, for skin tone determination and other biometric measurements, making health devices more accessible and equitable across racial and ethnic subgroups will require the convergence of hardware and algorithms. Nevertheless, this would have to happen in tandem with standardization and documentation of measurement types used in research studies and prospective trials.

## Interaction of human factors and cultural practices

However, bias in medical devices is not limited to skin tone. Human factors and cultural practices can also contribute to unexpected consequences in the performance of medical technologies.

### Neurological diseases

EEG provides another example of additional sources of bias. Because the technology is inexpensive and offers high-quality spatial resolution, it is one of the most popular techniques in neuroscience research, neurology, and sleep medicine. In particular, EEG signals are used to diagnose and inform the treatment of brain and sleep disorders and diseases such as epilepsy, seizure(s), brain tumors, stroke, or brain inflammation. Nonetheless, neuroscience research may suffer from unintended racial biases due partly to using physical electrodes in EEG [51]. While both the adhesion of electrodes to the patient’s scalp and their correct placement are essential to obtain neural responses from the scalp into the EEG sensors’ electrodes, suboptimal adhesion and placement can introduce noise and dampen signal amplitude [51,52].

**Fig 3:**
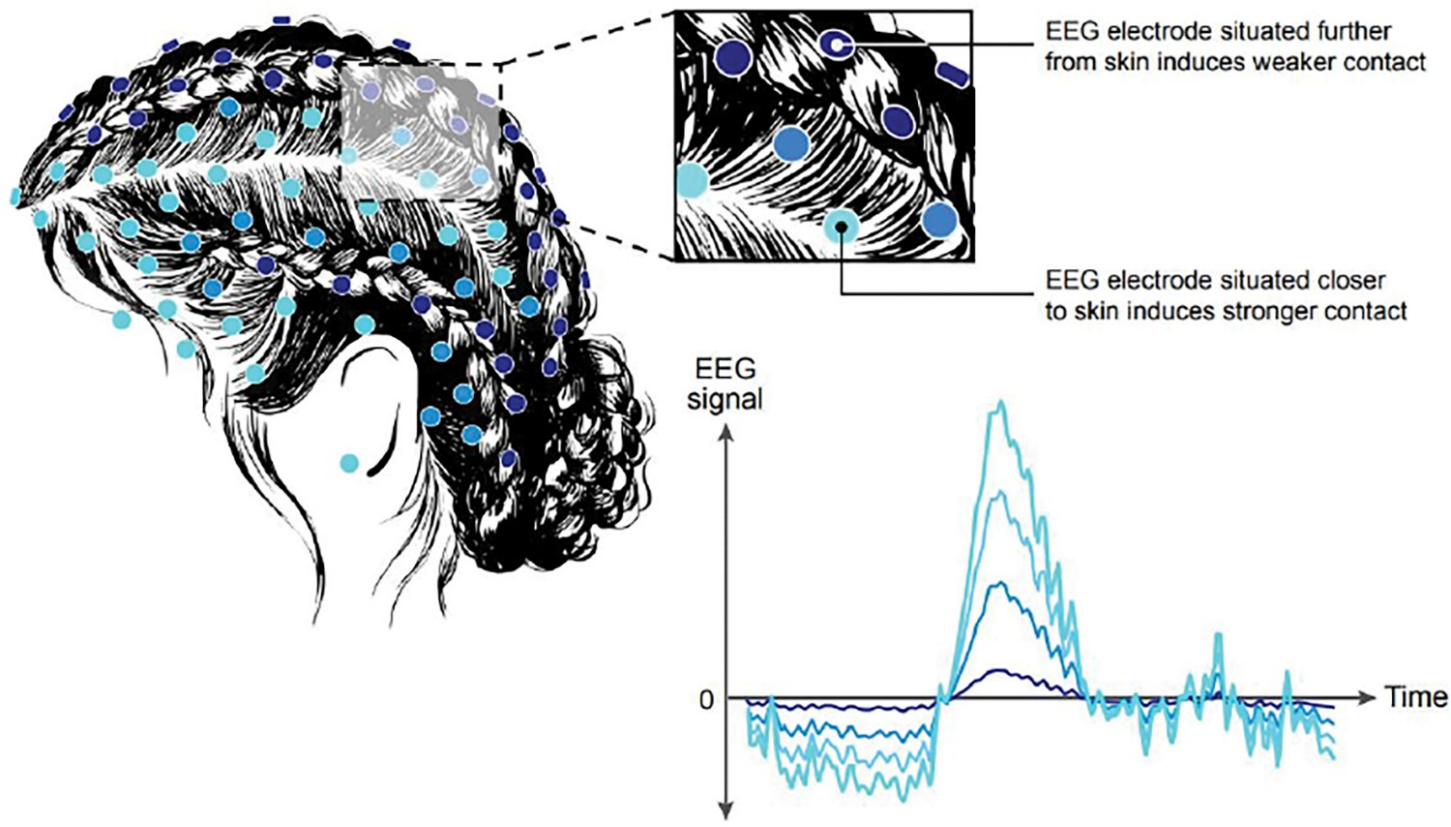
Adhesion of EEG electrodes at the surface of the scalp on areas with and without cornrows. EEG, electroencephalography.

Some studies have shown that Black hair is less likely to absorb liquid than Asian and Caucasian hair [53]. This may prevent the saline solution or conductive gel from acting as a proper conductor [51], thereby increasing the impedance of the sensor and ultimately reducing the quality of the neural response signals [51]. In practice, this artifact also brings cultural adaptation challenges and may result in lower participation of Black patients during data collection, as they might have to change their hairstyle (e.g., by removing cornrows or braids) to join the study [51]. Therefore, results from neuroscience studies can be more difficult to generalize to the Black patient population and extrapolation based on their Asian or Caucasian counterparts may be problematic (Fig 3).

Recent research [54] aims to improve the design of hair clips and electrodes to ensure their compatibility with a wider variety of hairstyles. However, newer prototypes present some pitfalls: for example, they have fewer than 128 channels on the skull, the current standard in EEG research. The bias is not solely due to sensor density, as skin conductance levels are significantly lower in Black patients than in their Asian and Caucasian counterparts [55]. Beyond electrophysiology, other brain recording techniques may also be affected by racial and ethnic biases. For example, functional near-infrared spectroscopy (fNIRS) can be impaired when performed on patients with dark hair because this technology relies on shining infrared light, measuring its reflection with optrodes to quantify blood flow in the brain [56]. All recommendations for EEG have been summarized in Table 3.

**Table 3:** Summary for electroencephalogram recommendations.

Technology	Recommendation	Strength	Level of evidence
EEG	Clinical: Patients’ hair and hairstyles can interact with EEG electrode adhesion and placement and may affect signal amplitude.	B	III
	Research: Diversity in hair and hairstyles should be considered in EEG and fNIRS research. Conversely, consider using MEG or OPM-MEG.	E	V

EEG, electroencephalography; fNIRS, functional near-infrared spectroscopy; MEG, magnetoencephalography; OPM-MEG, optically pumped magnetometer-MEG.

## Interpretation bias

Finally, even when medical devices generate unbiased data, their interpretation can be biased. The case of PFTs (spirometry) and examples taken from ophthalmology illustrate issues related to such *data interpretation bias*.

### Pulmonary medicine and spirometry

The perception that the lungs of certain patients are inherently inferior as a function of their race and ethnicity has been built into American medical practice since Samuel Cartright and Benjamin Gould [57]. PFTs assess lung function by measuring spirometry (i.e., airflow), lung volumes, and tissue function. Several components, including spirometry, involve correction factors, e.g., age, height, sex, and race. Normal ranges for PFTs are derived from vast samples such as the National Health and Nutrition Examination Survey (NHANES) or the Global Lung Initiative (GLI), which are supposed to be representative of the *national* population. Therefore, the corresponding lower and upper bounds are not *patient- or subgroup-specific*; instead, they only reflect aggregate measures emanating from surveys and represent *population-level* admissible values. Nevertheless, for such reference ranges to be useful in clinical practice, baseline populations must accurately characterize and reflect every patient. For example, although the patient cohort underlying the GLI PFT included black Americans, the reference equations in use were not appropriately rendering heterogeneity across the African continent and poorly fitted West African patients [58].

**Fig 4:**
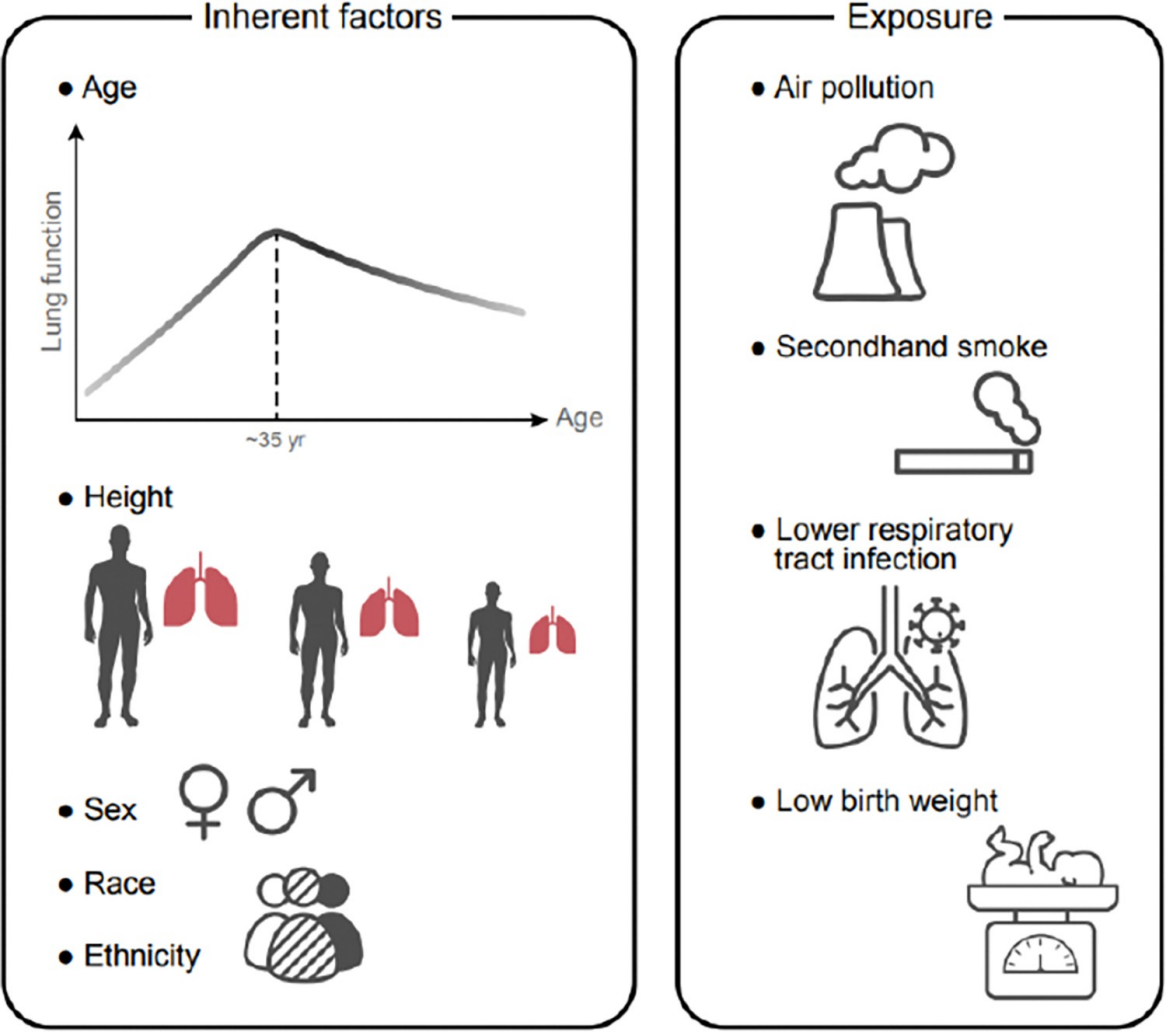
Impact of inherent factors versus exposure on lung function. The first graph notes that intrinsic lung function is affected by age and height. From birth, our lungs grow until our mid-30s, after which they slowly decline over time. Lung growth is affected by other factors than age. Height directly affects lung volume, as it ties into overall chest cavity volume. Sex similarly affects chest cavity volume. Race and ethnicity may be associated. Finally, “maximum potential” lung function is altered by exposure to air pollution, secondhand smoke, and lower respiratory tract infection: they would either reduce maximal potential (if experienced early in life) or increase the rate of decline (if experienced after maximal growth has occurred).

In addition to inherent racial and ethnic differences, studies suggest that socioeconomic factors and environmental exposures can significantly alter lung function. Indeed, existing biases result from both *inherent* and *acquired* factors [59]. For example, not only low birth weight, secondhand smoke, and air pollution are all associated with slower lung growth and lower lung function at an adult age [60,61]. Consequently, the range of *normal* values in the population may be affected by acquired influences such as community-level environmental and behavioral health factors, and the *true* normal may be higher than currently presumed (Fig 4).

However, PFT ranges are commonly used to determine the presence of disease. Because certain racial-ethnic subgroups can map to negative adjustment factors, a given low PFT value can be classified as *abnormal* in White patients but as *normal* in Black patients [61]. In addition to race and ethnicity, age plays an important role in the estimation of PFT ranges. At the transition between pediatric to adulthood (classically 18 years of age), using both pediatric and adult reference equations on the same patient could yield results varying by −14% to 38%, making their interpretation and subsequent decision-making difficult [62]. Age can further compound with race and ethnicity—among older patients from less adequately represented subgroups, e.g., older Asian patients, GLI equations were found to be less accurate than in other subgroups [63–65]. Therefore, determinations based on a single, nonadaptive threshold may negatively affect a broad array of clinical decisions—ranging from the prescription of medications [66] to the timely recognition of occupational lung disease [67], disease workup, and management [68]. More recently, inaccurate PFT interpretations could also impact patients recovering from COVID-19 disease, from being diagnosed with secondary pulmonary fibrosis to receiving pulmonary rehabilitation [69]. All recommendations for PFTs have been summarized in Table 4.

**Table 4:** Summary for PFT recommendations.

Technology	Recommendation	Strength	Level of evidence
PFTs	Clinical: Clinicians should be cautious in ensuring that the appropriate reference standard matches their patient. For example, the GLI PFT references for black Americans may poorly fit patients from West African nations.	B	II
	Research: More research is needed to understand the balance between sufficiently relevant and granular populations to apply to the patient while being broad enough to capture a clinically relevant definition of normal ranges.	E	V

GLI, Global Lung Initiative; PFT, pulmonary function test.

### Ophthalmology: Optical coherence tomography and Humphrey visual fields

New digital technologies have transformed ophthalmology. Some are already widely used in the clinical management of many diseases, including glaucoma and retinal diseases. Such procedures have yielded rich longitudinal, high-resolution databases containing clinical, imaging, and diagnostic testing information. However, biases in the underlying data generating processes can impact the clinical interpretation of the results and retrospective analyses conducted using these databases. Ophthalmologic technology offers 2 examples illustrative of such biases: optical coherence tomography (OCT) and Humphrey visual field (HVF) testing.

OCT technology is used to measure and map anatomical structures of the retina and optic nerve. The definition of *normal* and *abnormal* in OCT machines varies by manufacturer [70]. Each patient’s measurements are compared to population-wide distributions derived from testing several hundred normal patients usually selected from the country where the machine was manufactured. As a result, the population used to determine norms varies by machine. Its characteristics may differ from those of the population in which it will be used for clinical care, making the direct transfer of the technology—without intermediate recalibration—potentially biased.

Epidemiological studies show that OCT norms such as the thicknesses of the retina and nerve fiber layer (NFL) can vary by age [71,72], biological sex [73–75], race [72,75], and ethnicity [71,76,77]. Thinning from these NFL standards can be used to diagnose glaucoma and monitor progression [78]. However, a study of NFL thickness in a multiethnic Asian population revealed that it was 7.5 microns (approximately 7.7%) lower in Indian patients than in Malay or Chinese patients [79]. The authors report that this difference can adversely impact the sensitivity and specificity of glaucoma detection. They recommend refining OCT norms to reflect these ethnic but not racial differences.

Similarly, peripapillary capillary density, an OCT angiographic metric used to diagnose glaucoma, varies by race [80]. Patients of African descent have a lower peripapillary capillary density than patients of European descent, thus altering the sensitivity of glaucoma detection in patients of African descent, relative to patients of European descent.

HVF testing measures the sensitivity of the visual field and reveals defects which may occur in glaucoma and many other ophthalmologic conditions. The results of HVF testing can vary by age [81], biological sex [82], and race [72,73]—factors thought to be clinically relevant to diagnostic sensitivity. For example, longitudinal HVF test-retest measurements in patients with glaucoma show higher variability in patients of African descent than in those of European descent. This increased variability induces higher uncertainty in clinical decision-making, with modeling studies indicating delays in the diagnosis of glaucoma by up to 3 years among patients of African descent [73].

Addressing biases in ophthalmologic technologies will require collecting more granular sources of data from as many countries as possible to allow for input on norms and covariates from all segments of humanity [83]. All recommendations for OCT and HVF have been summarized in Table 5.

**Table 5:** Summary for OCT and HVF testing recommendations.

Technology	Recommendation	Strength	Level of evidence
OCT	Clinical: Using NFL thickness and peripapillary capillary density measurements for glaucoma diagnosis via OCT devices without having norms be validated in the population being studied (e.g., race, ethnicity, sex, and age) may affect sensitivity and specificity. Correlate clinically.	B	IV
	Research: Research reliant on NFT and peripapillary capillary density should validate reference norms across race and ethnicity.	E	V
HVF testing	Clinical: HVF testing criteria for glaucoma in longitudinal test-retest measurements may exhibit significant variability by age, sex, and race and delay diagnosis.	B	IV
	Research: Research using HVF criteria should validate reference norms across age, sex, and race.	E	V

HVF, Humphrey visual field; NFL, nerve fiber layer; NFT, nerve fiber thickness; OCT, optical coherence tomography.

## Discussion

This review has highlighted the different sources of potential bias in selected medical diagnostics, including physical, biological, and interpretation bias. For the convenience, a summary of all recommendations is highlighted in Table 6. Such biases can stem from flawed inclusion criteria, product design, or device validation.

Improving technologies to eliminate bias needs to occur at all levels. Researchers and manufacturers must develop unbiased technologies from the initial stages of their system design, whether in the choice of wavelengths and imaging methods for tools involving the patient’s skin, in the calibration of clinical equations or medical devices, or in the selection of their patient cohort. Finally, patients and even more so clinicians should be more alert to biases that exist in current technologies to avoid misinterpretation of results that may in turn lead to misdiagnosis. Furthermore, the role of industrial partners is not negligible. Health technology companies should increasingly adopt corporate social responsibility practices to incorporate ethics into their research practice and product design, thereby mitigating bias. Globally, it is crucial that organizations and policymakers worldwide, including the World Health Organization within the United Nations system, the European and African Unions, and the Association of Southeast Asian Nations (ASEAN), continue working toward universal health coverage and promoting the development of technologies that work for all types of individuals.

The feasibility of the solutions proposed in this narrative review highly depends on the countries involved. For example, regulation from the FDA could force US companies to develop less biased technologies—based on prespecified thresholds and device performance metrics—for their product to be marketed. However, applying the same regulations in Europe might be more challenging since consensus among European Union member states and coordination of the legal arsenal through the European Commission and partner organizations would be required. More importantly, developing a stronger regulatory framework can be a double-edged sword in countries with limited access to medical devices but whose populations are more prone to the biases described above.

### Future work and raising awareness

This review focused on selected domains that use BioMeTs with evidence of bias. For example, we did not mention wearable devices when discussing skin tone-related biases due to a lack of consensus in the research community and the need for larger trials to inform policy [50,84]. According to a study by Bent and colleagues [85], the accuracy of PPG heart rate measurements in selected wearable devices does not differ significantly across skin tones. However, the research-grade wearable device evaluated in the study is less accurate than consumer-grade devices when patients are at rest. Further, the technology relies on light absorption and uses primarily the green wavelengths, which are also absorbed by melanin. Therefore, biases can still emerge with new or homemade wearable devices and thus continued vigilance and evaluation is needed.

Moreover, we focused on domains with clinical utility. Biases also arise in other areas of biology and medicine. For example, analyses performed in genetics often use a reference genome obtained from averaging across individuals that do not reflect the diversity of the world’s population—the vast majority having European ancestry, thus making their application in other populations less effective [86]. Additionally, currently available genomic databases [87] still suffer from a lack of diversity, despite certain populations having higher genetic heterogeneity [88].

This narrative review is the first step toward identifying biases and inaccuracies in the medical technology we build and the clinical knowledge we generate. It is certainly not a complete review; instead, it should be considered as a compilation of examples illustrating how subtle omissions and considerations can result in a significant real-world impact on patients. As with pulse oximetry, research findings are often not rapidly translated into practical considerations for clinical care: Studies had documented the existence of discrepancies in estimated blood oxygen saturation measured by pulse oximeters among racial-ethnic subgroups long before the topic made the headlines. In addition to faster translational science, from the lab to the bedside, we must consistently reevaluate the practice of clinical care itself and revise how health services are being delivered.

### Limitations

Some of the studies discussed in this review, including research comparing body temperature measurements from NCIT and TAT devices, utilize convenience sampling methods to recruit participants. Thus, the resulting cohort of patients may not represent the target population, in which differences may ultimately be more significant than those reported in published work. In addition to limitations related to sampling methods, some studies have small sample sizes, such as Foglia and colleagues with 35 patients, which may limit the strength of conclusions [**2**]. As evidenced by other research groups, these publications can be inadequately used to justify the current state of affairs [16]. For example, there is a presumption of accuracy with insufficient statistical power when there may not be. We believe small studies should only be used to identify bias, but not to rule out the existence of bias.

Practical implementation of solutions to address the existing and documented digital determinants of health affecting medical devices that are already on the market will be a phased process. Fortunately, insufficient statistical power is no longer an issue for certain technologies, e.g., pulse oximetry. Yet, in light of recent publications [13–15], the next challenge for these technologies is to develop equivalent performance across all populations. As new digital products are being developed, research labs and manufacturers alike should exercise caution and be proactive to mitigate the potential for bias, both by determining optimal trial sample sizes and seeking sufficient representation in patient cohorts.

There is significant debate regarding the use of studies based on observational data to identify the presence of bias. For example, in his letter to the Orange County Business Journal [16], Joe Kiani, the CEO of Masimo, states that some studies contradict internal Masimo calibration data. However, device calibration data as measured by manufacturers are often kept private and not openly accessible to external research teams investigating sources of bias affecting medical technologies. Going forward, enforcing the release of tests conducted to assess device performance would help build trust in their reliability across patient subgroups.

Finally, device manufacturers should follow data interoperability standards (e.g., DICOM, Open mHealth, HL7 FHIR), unrestricted downloading capabilities, and harmonized data formatting to facilitate patient data acquisition and research that will help practitioners understand and mitigate potential biases in those they care for.

## Recommendations

Table 6 summarizes recommendations and assesses both strength and level of evidence for all technologies reviewed in this manuscript.

**Table 6:** Recommendations as per AHRQ levels of evidence [89].

Technology	Recommendation	Strength	Level of evidence
Pulse oximetry	Clinical: Pulse oximetry may be less accurate, especially as oxygen saturations decrease and in patients of color. Consider confirmatory testing with arterial blood gas and potentially modifying oxygen therapy.	B	III
	Clinical: Interventions requiring specific oxygen thresholds may need to be reevaluated, potentially across skin tones.	E	V
	Research: Prospective trials should incorporate sufficient diversity among participants to identify differential rates of deleterious outcomes, such as patient status deterioration, increased length of stay, or mortality.	E	V
	Research: Outcomes defined using pulse oximetry in research trials may need another method to measure oxygenation.	E	V
NCIT	Clinical: Use of NCITs may demonstrate wider variability in temperatures above 37.5°C, among patients with lighter skin pigmentation and among females. Correlate clinically or with another modality.	B	III
	Research: Menstrual cycles are associated with substantial temperature variations in females. Further research in females should consider the effect of these temperature variations on clinical decision thresholds.	E	V
Electroencephalogram	Clinical: Patients’ hair and hairstyles can interact with EEG electrode adhesion and placement and may affect signal amplitude.	B	III
	Research: Diversity in hair and hairstyles should be considered in EEG and fNIRS research. Conversely, consider using MEG or OPM-MEG.	E	V
PFT	Clinical: Clinicians should be cautious in ensuring that the appropriate reference standard matches their patient. For example, the GLI PFT references for black Americans may poorly fit patients from West African nations.	B	III
	Research: More research is needed to understand the balance between sufficiently relevant and granular populations to apply to the patient while being broad enough to capture a clinically relevant definition of normal ranges.	E	V
OCT	Clinical: Using nerve fiber layer thickness and peripapillary capillary density measurements for glaucoma diagnosis via OCT devices without having norms validated in the population being studied (e.g., race, ethnicity, sex, and age) may affect sensitivity and specificity. Correlate clinically.	B	IV
	Research: Research reliant on OCT NFT and peripapillary capillary density should validate reference norms across race and ethnicity.	E	V
HVF testing	Clinical: HVF testing criteria for glaucoma in longitudinal test-retest measurements may exhibit significant variability by age, sex, and race and delay diagnosis.	B	IV
	Research: Research using HVF criteria should validate reference norms across age, sex, and race.	E	V

**AHRQ Levels of Evidence** [89]

**Grade of research**:

A—Strongly recommend; good evidence.

B—Recommend; at least fair evidence

C—No recommendation for or against; balance of benefits and harms too close to justify a recommendation.

D—Recommend against; fair evidence is ineffective or harm outweighs the benefit.

E—Evidence is insufficient to recommend for or against routinely; evidence is lacking or of poor quality; benefits and harms cannot be determined.

**Level of evidence**:

Level I—Meta-analysis of multiple studies.

Level II—Experimental studies.

Level III—Well-designed, quasi-experimental studies.

Level IV—Well-designed, non-experimental studies.

Level V—Case reports and clinical examples.

EEG, electroencephalography; fNIRS, functional near-infrared spectroscopy; GLI, Global Lung Initiative; HVF, Humphrey visual field; NCIT, non-contact infrared thermometry; NFT, nerve fiber thickness; MEG, magnetoencephalography; OCT, optical coherence tomography; OPM-MEG, optically pumped magnetometer-MEG; PFT, pulmonary function test.

## Conclusion

In this review, we shed light on evidence of digital determinants of health in medical technologies and devices that do not rely on artificial intelligence and explore solutions to overcome these biases. In addition to differences in skin tones and light-based technologies, biases can arise from a lack of diversity in the composition of patient cohorts or from other physical characteristics. Future research should identify sources of bias left undetected to date, better document existing biases, and seek solutions to make health technologies and devices more accessible and accurate across all population groups. It is also critical to raise awareness about existing biases among researchers, practitioners, and policymakers in order to prevent the emergence of new types of bias.

## References

[1] Chidambaram S, Jain B, Jain U, Mwavu R, Baru R, et al. (Forthcoming, 2023) An introduction to digital determinants of health. PLOS Digital Health. 10.1371/journal.pdig.0000346 PMC10766177 38175828

[2] Foglia EE, Whyte RK, Chaudhary A, Mott A, Chen J, Propert KJ, et al. The Effect of Skin Pigmentation on the Accuracy of Pulse Oximetry in Infants with Hypoxemia. J Pediatr. 2017;182:375–377.e2. doi: 10.1016/j.jpeds.2016.11.043 27939107 PMC5328979

[3] Obermeyer Z, Powers B, Vogeli C, Mullainathan S. Dissecting racial bias in an algorithm used to manage the health of populations. Science. 2019;366:447–453. doi: 10.1126/science.aax2342 31649194

[4] Kadambi A. Achieving fairness in medical devices. Science. 2021;372:30–31. doi: 10.1126/science.abe9195 33795446

[5] Alba AC, Agoritsas T, Walsh M, Hanna S, Iorio A, Devereaux PJ, et al. Discrimination and Calibration of Clinical Prediction Models: Users’ Guides to the Medical Literature. JAMA. 2017;318:1377–1384. doi: 10.1001/jama.2017.12126 29049590

[6] Goldsack JC, Coravos A, Bakker JP, Bent B, Dowling AV, Fitzer-Attas C, et al. Verification, analytical validation, and clinical validation (V3): the foundation of determining fit-for-purpose for Biometric Monitoring Technologies (BioMeTs). NPJ Digit Med. 2020;3:55. doi: 10.1038/s41746-020-0260-4 32337371 PMC7156507

[7] Baxter CD, Waylonis GW. Therapeutic Lasers: Theory and Practice. Am J Phys Med Rehabil. 1995;74:327. Available from: https://journals.lww.com/ajpmr/citation/1995/07000/therapeutic_lasers__theory_and_practice.13.aspx.

[8] Feiner JR, Severinghaus JW, Bickler PE. Dark skin decreases the accuracy of pulse oximeters at low oxygen saturation: the effects of oximeter probe type and gender. Anesth Analg. 2007;105:S18–23, tables of contents. doi: 10.1213/01.ane.0000285988.35174.d9 18048893

[9] Bickler PE, Feiner JR, Severinghaus JW. Effects of skin pigmentation on pulse oximeter accuracy at low saturation. Anesthesiology. 2005;102:715–719. doi: 10.1097/00000542-200504000-00004 15791098

[10] Bickler PE, Feiner JR, Lipnick MS, Batchelder P, MacLeod DB, Severinghaus JW. Effects of Acute, Profound Hypoxia on Healthy Humans: Implications for Safety of Tests Evaluating Pulse Oximetry or Tissue Oximetry Performance. Anesth Analg. 2017;124:146–153. doi: 10.1213/ANE.0000000000001421 27529318

[11] Ries AL, Farrow JT, Clausen JL. Accuracy of two ear oximeters at rest and during exercise in pulmonary patients. Am Rev Respir Dis. 1985;132:685–689. doi: 10.1164/arrd.1985.132.3.685 4037542

[12] Ries AL, Prewitt LM, Johnson JJ. Skin color and ear oximetry. Chest. 1989;96:287–290. doi: 10.1378/chest.96.2.287 2752811

[13] Sjoding MW, Dickson RP, Iwashyna TJ, Gay SE, Valley TS. Racial Bias in Pulse Oximetry Measurement. N Engl J Med. 2020;383:2477–2478. doi: 10.1056/NEJMc2029240 33326721 PMC7808260

[14] Wong AI, Charpignon M, Kim H, Josef C, de Hond AAH, Fojas JJ, et al. Analysis of discrepancies between pulse oximetry and arterial oxygen saturation measurements by Race/Ethnicity and association with organ dysfunction and mortality. JAMA Netw Open. 2021. doi: 10.1001/jamanetworkopen.2021.31674 34730820 PMC9178439

[15] Henry NR, Hanson AC, Schulte PJ, Warner NS, Manento MN, Weister TJ, et al. Disparities in Hypoxemia Detection by Pulse Oximetry Across Self-Identified Racial Groups and Associations With Clinical Outcomes. Crit Care Med. 2022;50:204–211. doi: 10.1097/CCM.0000000000005394 35100193 PMC9070439

[16] Pulse oximeters are not racist. In: Orange County Business Journal [Internet]. 15 Feb 2021 [cited 2022 Jun 21]. Available from: https://www.ocbj.com/healthcare/pulse-oximeters-not-racist/.

[17] Center for Devices, Radiological Health. Pulse Oximeters—Premarket Notification Submissions [510(k)s]. 2020 [cited 2021 Apr 2]. Available from: https://www.fda.gov/regulatory-information/search-fda-guidance-documents/pulse-oximeters-premarket-notification-submissions-510ks-guidance-industry-and-food-and-drug.

[18] Center for Devices, Radiological Health. Pulse Oximeter Accuracy and Limitations. 2021 [cited 2021 Mar 22]. Available from: https://www.fda.gov/medical-devices/safety-communications/pulse-oximeter-accuracy-and-limitations-fda-safety-communication.

[19] Minasian LM, Carpenter WR, Weiner BJ, Anderson DE, McCaskill-Stevens W, Nelson S, et al. Translating research into evidence-based practice: the National Cancer Institute Community Clinical Oncology Program. Cancer. 2010;116:4440–4449. doi: 10.1002/cncr.25248 20572032 PMC2945622

[20] Dimond EP, St Germain D, Nacpil LM, Zaren HA, Swanson SM, Minnick C, et al. Creating a “culture of research” in a community hospital: Strategies and tools from the National Cancer Institute Community Cancer Centers Program. Clin Trials. 2015;12:246–256. doi: 10.1177/1740774515571141 25691600 PMC4420772

[21] FDA Guidance on Clinical Trial Diversity. J Nucl Med. 2021;62:23N–24N. Available from: https://www.ncbi.nlm.nih.gov/pubmed/33334919. 33334919

[22] Hamel LM, Penner LA, Albrecht TL, Heath E, Gwede CK, Eggly S. Barriers to Clinical Trial Enrollment in Racial and Ethnic Minority Patients With Cancer. Cancer Control. 2016;23:327–337. doi: 10.1177/107327481602300404 27842322 PMC5131730

[23] Clark LT, Watkins L, Piña IL, Elmer M, Akinboboye O, Gorham M, et al. Increasing Diversity in Clinical Trials: Overcoming Critical Barriers. Curr Probl Cardiol. 2019;44:148–172. doi: 10.1016/j.cpcardiol.2018.11.002 30545650

[24] All of Us Research Program. In: All of Us Research Program | NIH [Internet]. 1 Jun 2020 [cited 2022 Jul 3]. Available from: https://allofus.nih.gov/.

[25] Vardaki MZ, Kourkoumelis N. Tissue Phantoms for Biomedical Applications in Raman Spectroscopy: A Review. Biomed Eng Comput Biol. 2020;11:1179597220948100. doi: 10.1177/1179597220948100 32884391 PMC7440735

[26] Ntombela L, Adeleye B, Chetty N. Low-cost fabrication of optical tissue phantoms for use in biomedical imaging. Heliyon. 2020;6:e03602. doi: 10.1016/j.heliyon.2020.e03602 32258463 PMC7096755

[27] Edington CD, Chen WLK, Geishecker E, Kassis T, Soenksen LR, Bhushan BM, et al. Interconnected Microphysiological Systems for Quantitative Biology and Pharmacology Studies. Sci Rep. 2018;8:4530. doi: 10.1038/s41598-018-22749-0 29540740 PMC5852083

[28] Mishra D, Priyadarshini N, Chakraborty S, Sarkar M. Blood Oxygen Saturation Measurement Using Polarization-Dependent Optical Sectioning. IEEE Sens J. 2017;17:3900–3908. doi: 10.1109/JSEN.2017.2698520

[29] Jakachira R, Diouf M, Toussaint KC Jr. Rapid blood-oxygenation-saturation measurement using radially polarized light from light-emitting diodes. Biophotonics in Exercise Science, Sports Medicine, Health Monitoring Technologies, and Wearables III SPIE; 2022. p. 70–74. doi: 10.1117/12.2608805

[30] Chen W. Thermometry and interpretation of body temperature. Biomed Eng Lett. 2019;9:3–17. doi: 10.1007/s13534-019-00102-2 30956877 PMC6431316

[31] Khan S, Saultry B, Adams S, Kouzani AZ, Decker K, Digby R, et al. Comparative accuracy testing of non-contact infrared thermometers and temporal artery thermometers in an adult hospital setting. Am J Infect Control. 2021;49:597–602. doi: 10.1016/j.ajic.2020.09.012 33017627 PMC7530626

[32] Center for Devices Radiological Health. Non-contact Temperature Assessment Devices During the COVID-19 Pandemic. In: U.S. Food and Drug Administration [Internet]. FDA; [cited 2022 Jun 14]. Available from: https://www.fda.gov/medical-devices/coronavirus-covid-19-and-medical-devices/non-contact-temperature-assessment-devices-during-covid-19-pandemic.

[33] Liu C-C, Chang R-E, Chang W-C. Limitations of forehead infrared body temperature detection for fever screening for severe acute respiratory syndrome. Infect Control Hosp Epidemiol. 2004;25:1109–1111. doi: 10.1086/502351 15636300

[34] Bitar D, Goubar A, Desenclos JC. International travels and fever screening during epidemics: a literature review on the effectiveness and potential use of non-contact infrared thermometers. Eurosurveillance. 2009. doi: 10.2807/ese.14.06.19115-en 19215720

[35] Hewlett AL, Kalil AC, Strum RA, Zeger WG, Smith PW. Evaluation of an infrared thermal detection system for fever recognition during the H1N1 influenza pandemic. Infect Control Hosp Epidemiol. 2011;32:504–506. doi: 10.1086/659404 21515982

[36] Center for Devices, Radiological Health. Enforcement policy for telethermographic systems during the Coronavirus disease 2019 (COVID-19) public health emergency. In: U.S. Food and Drug Administration [Internet]. FDA; [cited 2022 Jun 14]. Available from: https://www.fda.gov/regulatory-information/search-fda-guidance-documents/enforcement-policy-telethermographic-systems-during-coronavirus-disease-2019-covid-19-public-health.

[37] Bone RC, Balk RA, Cerra FB, Dellinger RP, Fein AM, Knaus WA, et al. Definitions for sepsis and organ failure and guidelines for the use of innovative therapies in sepsis. The ACCP/SCCM Consensus Conference Committee. American College of Chest Physicians/Society of Critical Care Medicine. Chest. 1992;101:1644–1655. doi: 10.1378/chest.101.6.1644 1303622

[38] Geneva II, Cuzzo B, Fazili T, Javaid W. Normal Body Temperature: A Systematic Review. Open Forum Infect Dis. 2019;6:ofz032. doi: 10.1093/ofid/ofz032 30976605 PMC6456186

[39] Bhavani SV, Wiley Z, Verhoef PA, Coopersmith CM, Ofotokun I. Racial Differences in Detection of Fever Using Temporal vs Oral Temperature Measurements in Hospitalized Patients. JAMA. 2022;328:885–886. doi: 10.1001/jama.2022.12290 36066526 PMC9449792

[40] Yanovich R, Ketko I, Charkoudian N. Sex Differences in Human Thermoregulation: Relevance for 2020 and Beyond. Phys Ther. 2020;35:177–184. doi: 10.1152/physiol.00035.2019 32293229

[41] Baker FC, Siboza F, Fuller A. Temperature regulation in women: Effects of the menstrual cycle. Temperature (Austin). 2020;7:226–262. doi: 10.1080/23328940.2020.1735927 33123618 PMC7575238

[42] Kaciuba-Uscilko H, Grucza R. Gender differences in thermoregulation. Curr Opin Clin Nutr Metab Care. 2001;4:533–536. doi: 10.1097/00075197-200111000-00012 11706289

[43] Zheng H, Tan J, Ma K, Meng W. Changes in RT-PCR test results and symptoms during the menstrual cycle of female individuals infected with SARS-CoV-2: Report of two cases. J Med Virol. 2021;93:541–545. doi: 10.1002/jmv.26275 32639581 PMC7361390

[44] Bashkatov AN, Genina EA, Tuchin VV. OPTICAL PROPERTIES OF SKIN, SUBCUTANEOUS, AND MUSCLE TISSUES: A REVIEW. J Innov Opt Health Sci. 2011;04:9–38. doi: 10.1142/S1793545811001319

[45] Tseng S-H, Bargo P, Durkin A, Kollias N. Chromophore concentrations, absorption and scattering properties of human skin in-vivo. Opt Express. 2009;17:14599–14617. doi: 10.1364/oe.17.014599 19687939 PMC2754563

[46] Tseng S-H, Grant A, Durkin AJ. In vivo determination of skin near-infrared optical properties using diffuse optical spectroscopy. J Biomed Opt. 2008;13:014016. doi: 10.1117/1.2829772 18315374 PMC2626348

[47] Everett JS, Budescu M, Sommers MS. Making sense of skin color in clinical care. Clin Nurs Res. 2012;21:495–516. doi: 10.1177/1054773812446510 22645403 PMC3465481

[48] Ly BCK, Dyer EB, Feig JL, Chien AL, Del Bino S. Research Techniques Made Simple: Cutaneous Colorimetry: A Reliable Technique for Objective Skin Color Measurement. J Invest Dermatol. 2020;140:3–12.e1. doi: 10.1016/j.jid.2019.11.003 31864431

[49] Skin tone research @ Google. [cited 2022 Aug 4]. Available from: https://skintone.google/.

[50] Colvonen PJ. Response To: Investigating sources of inaccuracy in wearable optical heart rate sensors. NPJ Digit Med. 2021;4:38. doi: 10.1038/s41746-021-00408-5 33637822 PMC7910598

[51] Choy T, Baker E, Stavropoulos K. Systemic Racism in EEG Research: Considerations and Potential Solutions. Affect Sci. 2022;3:14–20. doi: 10.1007/s42761-021-00050-0 36042782 PMC9383002

[52] Webb EK, Etter JA, Kwasa JA. Addressing racial and phenotypic bias in human neuroscience methods. Nat Neurosci. 2022;25:410–414. doi: 10.1038/s41593-022-01046-0 35383334 PMC9138180

[53] Franbourg A, Hallegot P, Baltenneck F, Toutain C, Leroy F. Current research on ethnic hair. J Am Acad Dermatol. 2003;48:S115–9. doi: 10.1067/mjd.2003.277 12789163

[54] Etienne A, Laroia T, Weigle H, Afelin A, Kelly SK, Krishnan A, et al. Novel Electrodes for Reliable EEG Recordings on Coarse and Curly Hair. bioRxiv. 2020. p. 2020.02.26.965202. doi: 10.1109/EMBC44109.2020.9176067 33019375

[55] Alexandra Kredlow M, Pineles SL, Inslicht SS, Marin M-F, Milad MR, Otto MW, et al. Assessment of skin conductance in African American and Non-African American participants in studies of conditioned fear. Psychophysiology. 2017;54:1741–1754. doi: 10.1111/psyp.12909 28675471 PMC5638680

[56] Wassenaar EB, Van den Brand JGH. Reliability of near-infrared spectroscopy in people with dark skin pigmentation. J Clin Monit Comput. 2005;19:195–199. doi: 10.1007/s10877-005-1655-0 16244841

[57] Gould BA. Investigations in the Military and Anthropological Statistics of American Soldiers. US Sanitary Commission; 1869. Available from: https://play.google.com/store/books/details?id=dzgOAAAAIAAJ.

[58] Masekela R, Hall GL, Stanojevic S, Sartorius B, MacGinty R, Saad HB, et al. An urgent need for African spirometry reference equations: the Paediatric and Adult African Spirometry study. Int J Tuberc Lung Dis. 2019;23:952–958. doi: 10.5588/ijtld.18.0442 31533886

[59] Haynes JM, Kaminsky DA, Stanojevic S, Ruppel GL. Pulmonary Function Reference Equations: A Brief History to Explain All the Confusion. Respir Care. 2020;65:1030–1038. doi: 10.4187/respcare.07188 32156791

[60] Harik-Khan RI, Fleg JL, Muller DC, Wise RA. The effect of anthropometric and socioeconomic factors on the racial difference in lung function. Am J Respir Crit Care Med. 2001;164:1647–1654. doi: 10.1164/ajrccm.164.9.2106075 11719304

[61] Elmaleh-Sachs A, Balte P, Oelsner EC, Allen NB, Baugh A, Bertoni AG, et al. Race/Ethnicity, Spirometry Reference Equations, and Prediction of Incident Clinical Events: The Multi-Ethnic Study of Atherosclerosis (MESA) Lung Study. Am J Respir Crit Care Med. 2022;205:700–710. doi: 10.1164/rccm.202107-1612OC 34913853 PMC12042908

[62] Quanjer PH, Hall GL, Stanojevic S, Cole TJ, Stocks J. Global Lungs Initiative. Age- and height-based prediction bias in spirometry reference equations. Eur Respir J. 2012;40:190–197. doi: 10.1183/09031936.00161011 22183491

[63] Abdullah N, Borhanuddin B, Shah SA, Hassan T, Jamal R. Global Lung Initiative 2012 spirometry reference values in a large Asian cohort of Malay, Chinese and Indian ancestry. Respirology. 2018;23:1173–1179. doi: 10.1111/resp.13330 29790229

[64] Hall GL, Cooper BG. Increasing diversity within the Global Lung Function Initiative. Respirology. 2018:1090–1091. doi: 10.1111/resp.13373 30024083

[65] Tian X-Y, Liu C-H, Wang D-X, Ji X-L, Shi H, Zheng C-Y, et al. Spirometric reference equations for elderly Chinese in Jinan aged 60–84 years. Chin Med J (Engl). 2018;131:1016–1022. doi: 10.4103/0366-6999.227840 29553052 PMC5937307

[66] Vestbo J, Hurd SS, Agustí AG, Jones PW, Vogelmeier C, Anzueto A, et al. Global strategy for the diagnosis, management, and prevention of chronic obstructive pulmonary disease: GOLD executive summary. Am J Respir Crit Care Med. 2013;187:347–365. doi: 10.1164/rccm.201204-0596PP 22878278

[67] Townsend MC. Spirometry in Occupational Health—2020. J Occup Environ Med. 2020;62:e208. doi: 10.1097/JOM.0000000000001851 32398505

[68] Raghu G, Collard HR, Egan JJ, Martinez FJ, Behr J, Brown KK, et al. An Official ATS/ERS/JRS/ALAT Statement: Idiopathic Pulmonary Fibrosis: Evidence-based Guidelines for Diagnosis and Management. Am J Respir Crit Care Med. 2011:788–824. doi: 10.1164/rccm.2009-040GL 21471066 PMC5450933

[69] Anderson MA, Malhotra A, Non AL. Could routine race-adjustment of spirometers exacerbate racial disparities in COVID-19 recovery? Lancet Respir Med. 2021;9:124–125. doi: 10.1016/S2213-2600(20)30571-3 33308418 PMC7867612

[70] Mehta N, Waheed NK. Diversity in optical coherence tomography normative databases: moving beyond race. Int J Retina Vitreous. 2020;6:5. doi: 10.1186/s40942-020-0208-5 32158551 PMC7057526

[71] Poon LY-C, Antar H, Tsikata E, Guo R, Papadogeorgou G, Freeman M, et al. Effects of Age, Race, and Ethnicity on the Optic Nerve and Peripapillary Region Using Spectral-Domain OCT 3D Volume Scans. Transl Vis Sci Technol. 2018;7:12. doi: 10.1167/tvst.7.6.12 30510856 PMC6262887

[72] Girkin CA, McGwin G Jr, Sinai MJ, Sekhar GC, Fingeret M, Wollstein G, et al. Variation in optic nerve and macular structure with age and race with spectral-domain optical coherence tomography. Ophthalmology. 2011;118:2403–2408. doi: 10.1016/j.ophtha.2011.06.013 21907415

[73] Stagg B, Mariottoni EB, Berchuck S, Jammal A, Elam AR, Hess R, et al. Longitudinal visual field variability and the ability to detect glaucoma progression in black and white individuals. Br J Ophthalmol. 2021. doi: 10.1136/bjophthalmol-2020-318104 33985963 PMC8589883

[74] Dai W, Tham Y-C, Chee M-L, Majithia S, Tan NYQ, Wong K-H, et al. Normative pattern and determinants of outer retinal thickness in an Asian population: the Singapore Epidemiology of Eye Diseases Study. Br J Ophthalmol. 2019;103:1406–1412. doi: 10.1136/bjophthalmol-2018-313159 30658991

[75] Wagner-Schuman M, Dubis AM, Nordgren RN, Lei Y, Odell D, Chiao H, et al. Race- and sex-related differences in retinal thickness and foveal pit morphology. Invest Ophthalmol Vis Sci. 2011;52:625–634. doi: 10.1167/iovs.10-5886 20861480 PMC3053303

[76] Kashani AH, Zimmer-Galler IE, Shah SM, Dustin L, Do DV, Eliott D, et al. Retinal thickness analysis by race, gender, and age using Stratus OCT. Am J Ophthalmol. 2010;149:496–502.e1. doi: 10.1016/j.ajo.2009.09.025 20042179 PMC2826608

[77] Nousome D, Mckean-Cowdin R, Richter GM, Burkemper B, Torres M, Varma R, et al. Retinal Nerve Fiber Layer Thickness in Healthy Eyes of Black, Chinese, and Latino Americans: A Population-Based Multiethnic Study. Ophthalmology. 2021;128:1005–1015. doi: 10.1016/j.ophtha.2020.11.015 33217471 PMC8128930

[78] DeLeón-Ortega JE, Arthur SN, McGwin G, Xie A, Monheit BE, Girkin CA. Discrimination between Glaucomatous and Nonglaucomatous Eyes Using Quantitative Imaging Devices and Subjective Optic Nerve Head Assessment. Invest Ophthalmol Vis Sci. 2006;47:3374–3380. doi: 10.1167/iovs.05-1239 16877405 PMC3882168

[79] Ho H, Tham Y-C, Chee ML, Shi Y, Tan NYQ, Wong K-H, et al. Retinal Nerve Fiber Layer Thickness in a Multiethnic Normal Asian Population: The Singapore Epidemiology of Eye Diseases Study. Ophthalmology. 2019;126:702–711. doi: 10.1016/j.ophtha.2018.11.031 30529130

[80] Moghimi S, Zangwill LM, Hou H, Wong B, Proudfoot J, Penteado RC, et al. Comparison of Peripapillary Capillary Density in Glaucoma Patients of African and European Descent. Ophthalmol Glaucoma. 2021;4:51–62. doi: 10.1016/j.ogla.2020.07.005 32693049 PMC7854768

[81] Rutkowski P, May CA. The peripheral and Central Humphrey visual field—morphological changes during aging. BMC Ophthalmol. 2017;17:127. doi: 10.1186/s12886-017-0522-3 28716057 PMC5514484

[82] Tan NYQ, Tham Y-C, Koh V, Cheung CY, Aung T, Wong TY, et al. The Effect of Gender on Visual Field Sensitivity: The Singapore Chinese Eye Study. Ophthalmic Epidemiol. 2019;26:183–188. doi: 10.1080/09286586.2019.1568505 30672362

[83] Nakayama LF, Kras A, Ribeiro LZ, Malerbi FK, Mendonça LS, Celi LA, et al. Global disparity bias in ophthalmology artificial intelligence applications. BMJ Health Care Inform. 2022;29. doi: 10.1136/bmjhci-2021-100470 35396248 PMC8996038

[84] Bent B, Enache OM, Goldstein B, Kibbe W, Dunn JP. Reply: Matters Arising “Investigating sources of inaccuracy in wearable optical heart rate sensors.” NPJ Digit Med. 2021;4:39. doi: 10.1038/s41746-021-00409-4 33637842 PMC7910441

[85] Bent B, Goldstein BA, Kibbe WA, Dunn JP. Investigating sources of inaccuracy in wearable optical heart rate sensors. NPJ Digit Med. 2020;3:18. doi: 10.1038/s41746-020-0226-6 32047863 PMC7010823

[86] Wong KHY, Ma W, Wei C-Y, Yeh E-C, Lin W-J, Wang EHF, et al. Towards a reference genome that captures global genetic diversity. Nat Commun. 2020;11:5482. doi: 10.1038/s41467-020-19311-w 33127893 PMC7599213

[87] Popejoy AB, Ritter DI, Crooks K, Currey E, Fullerton SM, Hindorff LA, et al. The clinical imperative for inclusivity: Race, ethnicity, and ancestry (REA) in genomics. Hum Mutat. 2018;39:1713–1720. doi: 10.1002/humu.23644 30311373 PMC6188707

[88] Campbell MC, Tishkoff SA. African genetic diversity: implications for human demographic history, modern human origins, and complex disease mapping. Annu Rev Genomics Hum Genet. 2008;9:403–433. doi: 10.1146/annurev.genom.9.081307.164258 18593304 PMC2953791

[89] St. Clair J. Table 1, AHRQ scale of research grades and levels. Agency for Healthcare Research and Quality (US); 2005.

